# Online learning and COVID-19: a meta-synthesis analysis

**DOI:** 10.6061/clinics/2020/e2286

**Published:** 2020-11-02

**Authors:** Cristina Pires Camargo, Patricia Zen Tempski, Fabio Freitas Busnardo, Milton de Arruda Martins, Rolf Gemperli

**Affiliations:** I Laboratorio de Cirurgia Plastica e Microgirurgia (LIM-04), Faculdade de Medicina (FMUSP), Universidade de Sao Paulo, SP, BR; II Faculdade de Medicina (FMUSP), Universidade de Sao Paulo, SP, BR; III Divisao de Cirurgia Plastica, Faculdade de Medicina (FMUSP), Universidade de Sao Paulo, SP, BR

**Keywords:** SARS-CoV-2, E-learning, Lockdown, Education, Medical

## Abstract

The COVID-19 pandemic demanded a quick shift from presential to e-learning processes. Unlike planned e-learning programs, medical schools have had to quickly deliver the entire medical curriculum using remote strategies. This study aimed to perform a meta-synthesis of previous pandemic situations and describe the experience of the São Paulo University School of Medicine.

We searched the Cochrane Central Register of Controlled Trials, Medline, EMBASE, Lilacs, Scopus, Web of Science, and ERIC, using the following keywords: (“SARS” OR “severe acute respiratory syndrome” OR “severe acute respiratory syndrome” OR “Middle East Respiratory Syndrome Coronavirus” OR “middle east respiratory syndrome*” OR “MERS-CoV” OR “Mers” OR “Middle Eastern Respiratory Syndrome*” OR “MERS-CoV*” OR “coronavirus” OR “Coronavirus Infections” OR “coronavirus*” OR “COVID-19” OR “2019-nCoV” OR “SARS-CoV-2”) AND (“online education” OR “Education, Distance” OR “e-learning” OR “course online” OR “flipped classroom”) AND (“lockdown” OR “social distance” OR “quarantine”). The endpoints were the online platforms used for online learning, the model of class, recorded *versus* online interaction, duration of online lectures, and students' and teachers' perceptions of online learning.

We retrieved 38 records; only seven articles studied online education methods related to the pandemic and social distancing rules. The most frequently used online platform was Zoom^®^. The studies examined both synchronous and asynchronous approaches. There was no evidence regarding duration and students’ and teachers' attitude.

This study suggests that the online learning shift was feasible; however, because of the nature of the education shift (pandemic), future studies must further analyze the educational structure.

## INTRODUCTION

Distance learning has been available for more than a century through printed manuals, and subsequently, at the beginning of the 20^th^ century, through radio, audio, and video tapes. However, only at the end of the 20^th^ century did the educational modality spread with the advent of the world wide web (WWW) (1).

Several models of online educational activities are available, with varied purposes: massive open online courses (MOOCs) (2), recorded classes, online live interaction, tutorials, short communications, and conferences. However, some students and teachers still resist adopting online teaching/learning modalities as a daily practice.

The COVID-19 pandemic has had a catalytic effect on the changes in educational processes worldwide (3,4). It caused an abrupt shift from face-to-face to online classes without enough time to plan and prepare virtual educational programs (5,6). Teachers restructured their educational plans and developed skills for teaching in a virtual environment while providing emotional support for their students (7).

As such, the pandemic has allowed students and teachers to get together in a classroom. Some questions arise: which online platform is more feasible to use; which model of class (synchronous or asynchronous model) is more effective; what is the best duration for online lectures; what are studentś and teachers’ perceptions of the online learning process? To answer these questions, we performed a meta-synthesis, and reported our experience at the São Paulo University School of Medicine between March 23 and June 23, 2020.

## MATERIAL AND METHODS

To assess available articles related to both online education and social distancing, we included in the search strategy terms related to other epidemic situations in the past (SARS and MERS). The search strategy used the following keywords:

Topic 1 - infection: “SARS” OR “severe acute respiratory syndrome” OR “severe acute respiratory syndrome” OR “Middle East Respiratory Syndrome Coronavirus” OR “middle east respiratory syndrome*” OR “MERS-CoV” OR “Mers” OR “Middle Eastern Respiratory Syndrome*” OR “MERSCoV*” OR “coronavirus” OR “Coronavirus Infections” OR “coronavirus*” OR “COVID-19” OR “2019-nCoV” OR “SARS-CoV-2”

Topic2-education: “online education” OR “Education, Distance” OR “e-learning” OR “course online” OR “flipped classroom”

Topic3-“lockdown” OR “social distance” OR “quarantine”

We searched the following databases up to April 2020:

1. Cochrane Central Register of Controlled Trials (CENTRAL) 2020, Issue 3, in the Cochrane Library, using the strategy presented in Appendix 1;
2. MEDLINE via Ovid (from 1946), using the strategy presented in Appendix 2;
3. Embase via Ovid (from 1974), using the strategy presented in Appendix 3;
4. LILACS (Latin American and Caribbean Health Science Information database, from 1982), using the strategy presented in Appendix 4;
5. Scopus, Elsevier’s citation tool (from 2004);
6. Web of Science/Web of Knowledge (Clarivate and Thomson Reuters) (from 1900);
7. Education Resources Information Center (ERIC) (from 1966);

and the following trial registries:

1. ISRCTN registry (http://www.isrctn.com);
2. ClinicalTrials.gov (http://www.clinicaltrials.gov);
3. Australian New Zealand Clinical Trials Registry (http://www.anzctr.org.au);
4. World Health Organization International Clinical Trials Registry Platform (ICTRP) (apps.who.int/trialsearch/); and
5. EU Clinical Trials Register( http://www.clinicaltrialsregister.eu\).

We also checked the bibliographies of the studies included for further references to relevant trials. We did not limit the search for study design, time, and language. We defined outcomes as (1) online platform used for online learning; (2) model of class, recorded *versus* online interaction (synchronous *versus* asynchronous); (3) duration of online lectures; and (4) students’ and teachers' perceptions of the online learning process.

The inclusion criterion was as follows: all studies that described distance learning in any epidemic/pandemic situation. Exclusion criteria were as follows: e-learning without any epidemic/pandemic context and studies that presented intervention for elementary and high school populations.

## RESULTS

We retrieved 38 records, and only seven articles studied online education methods related to pandemic and social distancing rules.

### Online platforms used for online learning

Three studies used more than one platform toll (7,8,9). One study did not show any information (2). Three studies used only one platform (10,11,12). All interaction platforms allowed the use of different devices (personal computers (PCs), smartphones, tablets).

### Model of class

Several studies recommended to use Google Meet and Zoom^®^ platforms for recorded or live interaction (13,14,15) (Table 2).

### Duration of online lectures

Only one study showed lecture durations (9). In this study, the average lecture duration was about one hour.

### Students' and teachers' perception of the online learning process

Regarding students’ perceptions, some articles showed a turning point in attitude toward student-centered learning (8). Soled et al. created a student task force to develop an organization that would optimize students' ability to help in the COVID-19 response (clinic, community, and serve as a liaison with the administration and hospital leaders to identify evolving needs and rapidly engage students in those efforts) (16).

Chinelatto et al. described the students’ perception of a medical program’s adaptation to remote learning (7). The graduation committee decided to interrupt educational activities for two weeks to give teachers time to reorganize their activities, outcomes, and educational strategies for the virtual environment. During this time, it was possible to train teachers to use digital tools and platforms, and search for students with difficulties connecting to online activities and offer computers and Internet access to them. In this period, the Center for Development of Medical Educational Development Center (CEDEM) promoted several meetings to discuss students' expectations and the emotional impact of quarantine. Meanwhile, the clerkship was responsible for first to fifth-year students and recorded their lectures at the University with professional support. The CEDEM implemented an online education schedule with synchronous and asynchronous interaction from the first to the fourth year of medical graduation. Many students were anxious about what they were missing out on because of the social distancing measures initiated as a result of COVID-19, while others understood this time as an opportunity to develop new competences.

## DISCUSSION

Our review searched through similar past endemic and epidemic situations to answer some questions about the educational process that has arisen in the present pandemic. The impact of agility and leadership capacity on implementing remote learning was clear.

Regarding the online platform, this systematic review showed Zoom and Google platforms as the most used online learning platforms. We adopted both at the São Paulo University.

Another important decision was the choice of recorded lectures and live interaction. As previously suggested, we adopted a mixed model (13,14,15). Live interaction is more dynamic than recorded lectures; however, sometimes we have to rely on recorded classes due to Internet connectivity problems and to avoid clerkship and student overload (14). The recorded model allows a pause and an adaptation in daily activities, which are strengths of online learning. Recorded lectures make flexible attendance possible. Some students reported that they prefer recorded lectures over live interaction, because it allows them the freedom to choose the best time to study (7).

There was not enough information in the literature about lecture duration. We observed some lectures divided into mini-lectures (20 minutes) to keep students focused, with one-minute questions or exercises between them. Case discussions, forums, projects, and portfolios were also used.

Students’ and teachers’ perceptions could be influenced by generational differences and personal technological abilities, and these perceptions can influence their satisfaction with the effectiveness of e-learning programs.

COVID-19 has had a catalytic effect in the shift to remote educational activities in medical training, breaking down some barriers and resistance from both teachers and students (17).

Nevertheless, online education is a convoluted endeavor in terms of a realistic understanding of compelling educational content delivery and participants' expectations. Online learning showed the advantage of using a student-centered model to facilitate educational access. On the other hand, in medical and other health professional courses, the main drawback is the impossibility to practice. In the future, we believe that a mixed model will be the most popular model for teaching in health professional undergraduate programs.

## CONCLUSION

The COVID-19 pandemic situation is more serious and has lasted more than the previous epidemic situations. For this reason, we did not have any previous experience of using it as a background. In conclusion, the e-learning shift is feasible. The pandemic situation requires a well-integrated trained team to detect students' and teachers' needs and provide prompt answers and support with digital tools. We are all surfing the virtual environment, with greater or less difficulty, and we have the firm conviction that education must not stop.

## AUTHOR CONTRIBUTIONS

Camargo CP worked in substantial contributions to the conception or design of the work; acquisition, analysis and interpretation of data for the work. Tempski PZ, Martins MA worked in substantial contributions to the conception or design of the work, critical review. Busnardo FF, Gemperli R contributed in interpretation of data for the work, and critical review.

## Figures and Tables

**Table 1:** Demographic characteristics of online experience during MERS and COVID epidemics.

Study ID	Country	Reason/Period	Medical field
Park 2016	South Korea	MERS/2016	Graduation (all medical fields)
Chick 2020	United States of America	COVID/2020	Surgery
Pather 2020	Australia/New Zealand	COVID/2020	Anatomy
Regier 2020		COVID/2020	Genetics
Zhou 2020b	China	COVID/2020	Emergency
Gonzales-Zamora 2020	United States of America	COVID/2020	Infectious diseases
Chinelatto 2020	Brazil	COVID/2020	Graduation (all medical fields)

**Table 2:** Description of online platform, model of class, accessibility and educational methods.

Study ID	Online platform	Online classes	Accessibility	Education methods
Park 2016	Skype	live	PCs/smartphones/tablets	Problem-based Learning
Chick 2020	Facebook (BSITE Daily)/ GoToMeeting (LogMeIn Inc., Boston, MA)	pre-recorded	PCs/smartphones/tablets	Flipped classroom and conferences
Pather 2020	Zoom		PCs/smartphones/tablets	Conferences
Regier 2020	Zoom	pre-recorded	PCs/smartphones/tablets	Case method
Zhou 2020b	no information			Micro-videos and questionnaires
Gonzales-Zamora 2020	Facebook/Zoom/Skype	pre-recorded	PCs/smartphones/tablets	Lectures and discussions
Chinelatto 2020	Google Meet/ Zoom	live pre-record	PCs/smartphones/tablets	Lectures, discussions in forum, texts, articles, and case discussions
